# The leading role of MYC in DNA damage response: exploring opportunities for therapeutic inhibition

**DOI:** 10.3389/fcell.2025.1726515

**Published:** 2026-01-05

**Authors:** Fabio Giuntini, Jonathan R. Whitfield, Daniel Massó-Vallés, Laura Soucek

**Affiliations:** 1 Models of Cancer Therapies Group, Vall d’Hebron Institute of Oncology (VHIO), Vall d’Hebron Barcelona Hospital Campus, Barcelona, Spain; 2 Peptomyc S.L., Vall d’Hebron Barcelona Hospital Campus, Barcelona, Spain; 3 Institució Catalana de Recerca i Estudis Avançats (ICREA), Barcelona, Spain; 4 Department of Biochemistry and Molecular Biology, Universitat Autònoma de Barcelona, Bellaterra, Spain

**Keywords:** combination (combined) therapy, DDR (DNA damage response), DDR inhibitors, DNA damage, MYC, MYC inhibition

## Abstract

MYC performs a dual role in DNA Damage Response (DDR), promoting genomic instability through replication stress, R-loop formation, and topoisomerase-mediated damage, while simultaneously activating DNA repair pathways to maintain cell survival. This review provides a comprehensive analysis of how MYC inhibition affects DDR pathway dependencies. In fact, when MYC is inhibited, cancer cells lose both their proficient DNA repair capacity and their protective mechanisms against replication stress. This creates a therapeutic window in which combining MYC inhibitors with DDR-targeting agents may achieve synergistic anti-cancer effects. Central to this approach is the exploration of rational combination strategies that pair MYC inhibitors with various DDR modulators including Poly (ADP-ribose) polymerase (PARP) inhibitors, ATR/CHK1 inhibitors, and other DNA repair pathway disruptors. This review summarizes preclinical evidence demonstrating enhanced therapeutic efficacy when MYC inhibition is combined with DDR-targeting agents and discusses early clinical findings that support this promising therapeutic strategy.

## 1 Introduction

MYC is a fundamental transcription factor involved in orchestrating numerous cellular processes critical for normal physiology (Zacarías-Fluck et al., 2024). However, besides being a main actor in diverse biological functions, MYC also holds a most infamous role in ruthless oncogenic transformation. Indeed, MYC is deregulated or overexpressed in over 70% of cancers, providing them with fundamental characteristics for their survival and maintenance (Dang, 2012; Llombart and Mansour, 2022).

Unlike many oncogenes that exhibit tissue-specific patterns of alteration, MYC is deregulated in both haematological malignancies and solid tumours through multiple mechanisms (Carroll et al., 2018). Another peculiarity is the fact that MYC is rarely mutated in cancers, where direct amplification of the MYC locus represents the most apparent form of its genetic alteration. Other frequent means of MYC deregulation are chromosomal translocations, enhanced mRNA stability, altered post-translational modifications, and dysregulation of upstream signalling pathways (Dang, 2012), reflecting the selective pressure on tumours to elevate MYC activity through whatever means available, reinforcing its critical role in malignant transformation and progression.

Importantly, the oncogenic potency of MYC stems directly from its hijacking of normal physiological functions, with cancer cells taking over these processes for malignant advantage. Indeed, MYC’s contributions to oncogenesis can be mapped directly onto Hanahan and Weinberg’s “Hallmarks of Cancer” (Hanahan, 2022; Hanahan and Weinberg, 2000; Hanahan and Weinberg, 2011), a conceptual framework that defines the acquired capabilities necessary for malignant transformation. Through its pleiotropic effects on cellular processes, MYC enables virtually all these hallmark capabilities (Llombart and Mansour, 2022).

**FIGURE 1:**
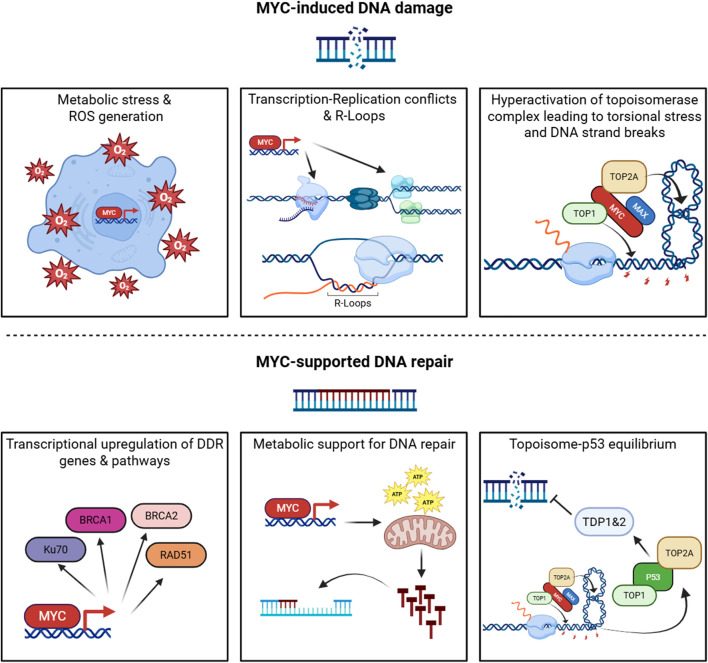
The dual role of MYC in the DNA damage response. MYC coordinates opposing functions that both generate and mitigate DNA damage. Upper panels (MYC-induced DNA damage): MYC drives metabolic and oxidative stress leading to ROS accumulation; promotes transcription- replication conflicts and R-loop formation; and enhances topoisomerase activity, Increasing torsional stress and DNA strand breaks. Lower panels (MYC-supported DNA repair): MYC upregulates key DNA damage response genes and pathways, provides metabolic support for nucleotide biosynthesis and repair, and modulates the topoisomerase p53 axis to maintain DNA repair equilibrium. “Topoisome” is used here to denote MYC-associated topoisomerase complexes (TOP1/TOP2) that regulate DNA topology during intense transcription. Created with BioRender.com.

Among its diverse oncogenic functions, MYC’s relationship with DNA damage represents one of the most intriguing and paradoxical aspects (Campaner and Amati, 2012). Indeed, MYC displays a dual nature in the DDR, where it simultaneously promotes genomic instability through replication stress (Herold et al., 2009) while enhancing DNA repair mechanisms via transcriptional activation of DDR genes (Luoto et al., 2010). Such opposing functions create an intricate balance that enables cancer cells to sustain elevated levels of genomic stress while maintaining survival (Dominguez-Sola and Gautier, 2014), positioning MYC as a complex transcription factor capable of performing contradictory roles in the same cellular context (Figure 1).

## 2 MYC as a promoter of genomic chaos

MYC multifaceted capacity to induce replication stress makes it key in the induction of genomic instability observed in cancer cells. MYC directly regulates genes involved in nucleotide metabolism and DNA replication, including components of the pre-replication complex (Figure 1) (Dominguez-Sola et al., 2007). By promoting accelerated cell cycle progression and origin firing without proportional increases in fork progression, MYC exacerbates replication fork stalling and collapse (Groh and Gromak, 2014). The mechanistic basis of this phenomenon has been further elucidated through studies demonstrating that MYC overexpression triggers premature S-phase entry and ectopic replication origin activation (Macheret and Halazonetis, 2018; Murga et al., 2011), creating a cellular environment where transcription and replication machineries are forced to compete for the same DNA template.

Indeed, MYC induces genomic instability through transcription–replication conflicts, where RNA polymerase II collides with the DNA polymerases (Figure 1). These machineries can clash in either codirectional or head-on orientations, with the latter being especially deleterious, resulting in replication fork stalling, R-loop accumulation, and DNA double-strand breaks (DSBs) (Garcia-Muse and Aguilera, 2016). The problem is amplified by MYC’s induction of exceptionally long transcriptional units and premature origin firing, which increases the probability of replication encountering an actively transcribing RNA polymerase II complex during S phase (Hamperl et al., 2017). Recent studies by the Eilers group added further nuance: MYC protein has been observed to multimerize at stalled replication forks (Solvie et al., 2022), and MYCN can recruit the nuclear exosome targeting complex to nascent RNAs leading to S phase progression and tumour cell stress resilience (Papadopoulos et al., 2024). These findings underscore the intensity of transcription–replication stress under MYC overexpression, where additional mechanisms are required to keep conflict-induced damage in check. Thus, transcription–replication collisions emerge as a defining source of MYC-driven genomic instability, setting the stage for the broader oncogene-induced DNA damage phenotype.

Beyond its indirect effects on replication machinery competition, MYC provokes DNA damage directly through enhanced “topoisome” activity. MYC interacts with topoisomerases TOP1, TOP2A, and TOP2B to form topoisome complexes that stimulate topoisomerase function at sites of active transcription (Das et al., 2022). While this mechanism initially evolved to alleviate torsional stress generated by intensive transcriptional activity, oncogenic MYC levels hyperactivate topoisome function, leading to excessive DNA strand breaks. Under conditions of replication stress or compromised repair capacity, these topoisomerase-mediated transient breaks can convert to permanent DNA lesions, directly contributing to the genomic instability that characterizes MYC-driven cancers (Figure 1) (Das et al., 2024).

These mechanisms collectively explain early studies demonstrating that even transient MYC overexpression could induce karyotypic abnormalities and promote transformation (Mai et al., 1996), and contribute to what Vafa et al. termed “oncogene-induced DNA damage,” wherein MYC activation generates persistent genomic instability. This instability, in turn, may facilitate the accumulation of mutations that drive cancer evolution and heterogeneity (Vafa et al., 2002).

The clinical significance of MYC-induced genomic instability extends beyond its role in initial transformation. Tumour cells expressing elevated MYC levels exist in a state of chronic replication stress, characterized by persistent activation of DNA damage checkpoints and elevated mutation rates. This creates a cellular environment where genomic instability becomes both a driver of malignant progression and a potential therapeutic vulnerability, as cancer cells become increasingly dependent on DNA repair pathways to survive the constant barrage of replication-associated damage (Kuzyk and Mai, 2014). In this way, MYC establishes itself not only as a relentless instigator of genomic instability but also as a paradoxical safeguard of genome integrity, as discussed in the next section.

## 3 MYC’s rescue role in DNA repair

Genomic analyses reveal that MYC regulates approximately 10% of the human genome, including key components of multiple DNA repair pathways (Dang, 2012). Luoto et al. demonstrated that MYC depletion sensitises cancer cells to DNA damage, suggesting that MYC-dependent repair pathways are essential for tumour cell survival following genotoxic stress (Luoto et al., 2010).

This transcriptional control extends to genes involved in homologous recombination (HR), non-homologous end joining (NHEJ), and base-excision repair (BER). For instance, MYC directly regulates expression of key HR components such as RAD51, BRCA1, and BRCA2 in multiple cancer contexts (Figure 1) (Luoto et al., 2010). Furthermore, MYC modulation of chromatin structure may facilitate repair protein access to DNA lesions, enhancing repair efficiency (Kuzyk and Mai, 2014).

Beyond direct effects on DNA damage and repair, MYC modulates DDR through broader cellular processes. MYC-driven metabolic reprogramming influences the availability of metabolites required for DNA repair, including nucleotides and SAM (S-adenosylmethionine) (Luo et al., 2010). Additionally, MYC-induced changes in cellular energetics may provide the ATP required for energy-intensive repair processes, further supporting cancer cell survival under genotoxic stress (Figure 1) (Morrish et al., 2008).

Work by Levens and colleagues has revealed another dimension to MYC’s role in DNA damage regulation through its direct interaction with DNA topoisomerases. While, as illustrated in the previous section, topoisome complexes contribute to genomic instability, these same complexes at physiological levels also serve protective functions, managing the torsional stress generated during high-intensity transcription and replication. Initial studies in this context demonstrated that MYC nucleates specialised topoisome complexes containing TOP1 and either TOP2A or TOP2B, depending on whether MYC or MYCN is involved (Das et al., 2022). By stimulating topoisomerase activity and increasing its presence at promoters, gene bodies, and enhancers, MYC helps maintain genomic function under conditions of elevated transcriptional output. More recent work from the same group has uncovered that excessive MYC-topoisome activity directly damages DNA, triggering ATM activation and p53 response (Das et al., 2024). This initiates a regulatory circuit wherein p53 forms its own distinct topoisomes (p53–TOP1–TOP2) that not only support p53 target gene expression but also induce repair enzymes TDP1 and TDP2 to mitigate topoisomerase-mediated DNA damage (Figure 1). This relationship between MYC- and p53-driven topoisomes represents yet another layer of MYC effect on DNA integrity, where MYC-induced damage activates counterbalancing protective mechanisms to prevent catastrophic genomic instability.

Checkpoint recovery and adaptation are central determinants of whether MYC-driven DNA damage becomes terminal. PLK1-mediated degradation of Claspin attenuates CHK1 signalling, promoting checkpoint termination and CDK1 reactivation, events that permit mitotic entry despite residual DNA lesions (De Blasio et al., 2019). Likewise, modulation of WEE1–CDK1 balance alters the threshold for mitotic progression. In MYC-high contexts, accelerated cell-cycle kinetics and modified checkpoint recovery can allow cells to tolerate greater DNA damage, fostering an environment where DDR inhibition may either cause catastrophic mitosis or be evaded by adaptation. Therefore, mechanistic interplay among PLK1, Claspin, WEE1 and CDK1 should be considered when predicting responses to MYC–DDR combination strategies (De Blasio et al., 2019; Verma et al., 2019).

As Campaner and Amati propose, these seemingly contradictory perspectives may represent two sides of the same coin: MYC appears to function as both a DNA damage inducer and a DDR activator, creating an “oncogene-induced DDR” (Campaner and Amati, 2012). This balanced system allows cancer cells to maintain sufficient genomic instability to facilitate advantageous mutations while preventing catastrophic damage that would trigger cell death. This apparent paradoxical relationship could be conceptualised as “genome instability within limits”, wherein MYC-driven cancers walk a precarious line between beneficial mutation and lethal genomic catastrophe and may partially explain the selective pressure for MYC deregulation across diverse cancer types (Maya-Mendoza et al., 2015).

## 4 Targeting DDR vulnerabilities in cancer

Cancer development and progression are associated with genomic instability, which stems from defects in the very systems designed to maintain genomic integrity. While the DDR evolved as a protective mechanism against genotoxic threats, cancer cells frequently present alterations in these pathways that initially provide them with a selective advantage but eventually can create exploitable therapeutic vulnerabilities (Hanahan and Weinberg, 2011).

Mutations in key DDR genes are frequently observed across cancer types, with notable examples including TP53, BRCA1/2, and ATM (Lord and Ashworth, 2012). These mutations not only contribute to cancer initiation but also influence treatment response and disease progression. The dysregulation of cell cycle checkpoints represents a critical mechanism through which cancer cells bypass normal DDR function. Approximately 50% of human cancers harbour TP53 mutations, compromising G1 checkpoint control and allowing cells with DNA damage to continue dividing (Kastenhuber and Lowe, 2017). Similarly, aberrations in the ATM-CHK2 and ATR-CHK1 pathways disrupt the S and G2/M checkpoints, respectively, further promoting genomic instability (Bartek and Lukas, 2003). The relationship between MYC-driven DDR gene expression and p53-mediated cell-fate decisions is shaped by well-established principles of p53 threshold dynamics. p53 activity is governed by the balance between DDR-driven activation (ATM/CHK2) and negative feedback via MDM2, as well as by regulators of checkpoint adaptation such as PLK1 and Claspin (Verma et al., 2019). These mechanisms determine whether p53 responds to persistent replication stress with temporary cell-cycle arrest and repair, or transitions into apoptosis when damage exceeds the repair capacity. In MYC-overexpressing cells, enhanced DDR gene transcription may help maintain p53 activity within a pro-survival range, whereas failure to resolve replication stress can shift this equilibrium toward cell-death pathways.

PARP enzymes are critical for DNA damage repair, particularly in the BER single-strand DNA repair pathway (Rouleau et al., 2010). PARP1, the most abundant member of the PARP family, recognises and binds to sites of DNA damage, catalysing the addition of poly (ADP-ribose) (PAR) chains to itself and other acceptor proteins (Gibson and Kraus, 2012). This post-translational modification, known as PARylation, facilitates the recruitment of DNA repair factors to the damaged sites (Ray Chaudhuri and Nussenzweig, 2017). PARP2, though less abundant, shares the ability to detect DNA damage and synthesize PAR chains but has distinct substrate preferences and binding patterns (Ali et al., 2016).

PARP inhibitors (PARPi) function by blocking the catalytic activity of PARP enzymes, primarily PARP1, thereby preventing the synthesis of PAR chains (Lord and Ashworth, 2016). This inhibition leads to the accumulation of unrepaired single-strand breaks (SSBs), which can convert to DSBs during DNA replication. In normal cells, these DSBs would be repaired through HR. However, in cells with defective HR, such as those harbouring mutations in BRCA1 or BRCA2, these DSBs remain unrepaired, leading to genomic instability and ultimately cell death (Farmer et al., 2005; Bryant et al., 2005). This therapeutic strategy exploits a concept known as synthetic lethality, where the simultaneous inactivation of two genes or pathways leads to cell death, whilst inactivation of either alone is compatible with cell survival (Kaelin, 2005). In the context of PARPi, synthetic lethality occurs between PARPiand HR deficiency (HRD) (Fong et al., 2009).

Beyond catalytic inhibition, PARPi also functions through PARP trapping, where PARP1 and PARP2 become trapped on DNA, forming cytotoxic PARP-DNA complexes that interfere with DNA replication (Pommier et al., 2016). This mechanism may contribute significantly to the cytotoxicity of PARPi, particularly in HRD cells, as PARP trapping leads to replication fork collapse and subsequent DSBs, which are normally repaired by HR. In the absence of functional HR, these DSBs remain unresolved, making trapped PARP-DNA complexes considerably more cytotoxic than unrepaired SSBs caused by PARP inactivation alone (Murai et al., 2012; Hopkins et al., 2015).

Several PARPi have received regulatory approval for clinical use, with varying indications and pharmacological properties, as described in Table 1. Olaparib, the first PARPi to receive US Food and Administration (FDA) approval in 2014, was initially indicated for germline BRCA-mutated advanced ovarian cancer patients with platinum-sensitive relapse (Ledermann et al., 2014).

**TABLE 1:** PARP inhibitors: clinical approvals and key trials.

PARP Inhibitor	Brand/research code name	Cancer type	Clinical setting/indication	Development status	Pivotal trial(s)	Key reference(s)
Olaparib	Lynparza	Ovarian cancer	gBRCA-mutated, platinum-sensitive relapse	Approved	Study 19 NCT00753545	Ledermann et al. (2014)
			gBRCA-mutated, first-line maintenance	Approved	SOLO1 NCT01844986	Moore et al. (2018)
			gBRCA-mutated, relapsed platinum-sensitive	Approved	SOLO2 NCT01874353	Pujade-Lauraine et al. (2017)
			HRD-positive, first-line (+ bevacizumab)	Approved	PAOLA-1 NCT02477644	Ray-Coquard et al. (2019)
		Prostate cancer (mCRPC)	BRCA1/2 or ATM-mutated, post-AR therapy	Approved	PROfound NCT02987543	de Bono et al. (2020)
		Pancreatic cancer	gBRCA-mutated metastatic, maintenance	Approved	POLO NCT02184195	Golan et al. (2019)
		Breast cancer (TNBC)	gBRCA-mutated, HER2-negative metastatic	Approved	OlympiAD NCT02000622	Robson et al. (2017)
Rucaparib	Rubraca	Ovarian cancer	gBRCA-mutated, high-grade	Accelerated Approval	Study 10 & ARIEL-2 NCT01482715 NCT01891344	Oza et al. (2017)
			BRCA-mutated/HRD-positive, platinum-sensitive	Approved	ARIEL3 NCT01968213	Coleman et al. (2017)
			First-line maintenance	Approved	ATHENA-MONO NCT03522246	Monk et al. (2022)
		Prostate cancer (mCRPC)	BRCA-mutated	Accelerated approval	TRITON2 NCT02952534	Abida et al. (2020)
		Prostate cancer (mCRPC)	BRCA-mutated	Approved	TRITON3 NCT02975934	Fizazi et al. (2023)
Niraparib	Zejula	Ovarian cancer	First-line maintenance (biomarker-agnostic)	Approved	PRIMA NCT02655016	Gonzalez-Martin et al. (2019)
			Recurrent, platinum-sensitive (biomarker-agnostic)	Approved	NOVA/PRIMA-1 NCT01847274	Mirza et al. (2016)
			Recurrent, platinum-sensitive	Approved	NORA NCT03705156	Wu et al. (2024b)
Talazoparib	Talzenna	Breast cancer (TNBC)	gBRCA-mutated, HER2-negative metastatic	Approved	EMBRACA NCT01945775	Litton et al. (2018)
Senaparib	Pamiray	Ovarian cancer	Platinum-sensitive recurrent	Approved (China)	FLAMES NCT04169997	Wu et al. (2024a)
Fluzoparib/Fuzuloparib	AiRuiYi	Ovarian cancer	gBRCA-mutated, platinum-sensitive recurrent	Approved (China)	FZOCUS-3 NCT03509636	Li et al. (2021)
			Maintainance therapy	Approved (China)	FZOCUS-2 NCT03863860	Li et al. (2022)
Pamiparib	BGB-290	Ovarian cancer	Platinum-sensitive recurrent	Approved (China)	BGB-290-102 NCT03333915	Wu et al. (2022)

Summary of PARP inhibitors approved or in clinical development, including their commercial names, cancer indications, regulatory status, and pivotal clinical trials. All approved agents have received regulatory authorization from the FDA (USA), EMA (EU), and/or NMPA (China). Key references cite the primary publication for each pivotal trial.

Following Olaparib, Rucaparib, Niraparib, Talazoparib and Senaparib have also received regulatory approval by the FDA, the European Medicines Agency (EMA) and/or other agencies. These agents differ in their PARP-trapping potency, selectivity for PARP1/2 versus other PARP family members, and pharmacokinetic profiles, which may influence their efficacy and toxicity in different clinical contexts (Lord and Ashworth, 2017).

In ovarian cancer, PARPi have become a cornerstone of maintenance therapy, with multiple agents approved based on pivotal Phase II/III trials. Olaparib gained approval following SOLO-1, which demonstrated a significant increase in Progression-Free Survival (PFS) in BRCA-mutated patients in the first-line setting (Moore et al., 2018), and SOLO-2, which confirmed efficacy in relapsed, platinum-sensitive BRCA-mutated cases (Pujade-Lauraine et al., 2017). The PAOLA-1 trial showed that combining Olaparib with Bevacizumab extended PFS in HRD-positive patients (Ray-Coquard et al., 2019), while Study 19 provided early evidence of clinical benefit of Olaparib monotherapy in relapsed disease (Ledermann et al., 2014). Niraparib showed broad efficacy in newly-diagnosed and recurrent platinum-sensitive disease regardless of biomarker status in the PRIMA (Gonzalez-Martin et al., 2019) and NOVA/PRIMA-1 (Mirza et al., 2016) and NORA trials (Wu et al., 2024b). Rucaparib received accelerated approval in 2016 based on the integrated analysis of Study 10 and ARIEL2, which demonstrated antitumor activity in BRCA-mutant high-grade ovarian cancer (Oza et al., 2017). It was subsequently approved in recurrent, platinum-sensitive ovarian cancer based on ARIEL3, which showed improved PFS in BRCA-mutant and HRD subgroups (Coleman et al., 2017), and its role was further expanded by the ATHENA trial in the first-line setting (Monk et al., 2022). In recent years, three PARPi have been approved by the China National Medical Products Administration (NMPA): Fluzoparib received its first approval based on the Phase 2 FZOCUS-3 trial in BRCA-mutant platinum-sensitive recurrent ovarian cancer (Li et al., 2021), and subsequently received approval for maintenance therapy based on the Phase 3 FZOCUS-2 trial (Li et al., 2022); Senaparib, which demonstrated improved PFS and manageable toxicity in platinum-sensitive recurrent disease in the FLAMES study (Wu et al., 2024a); and Pamiparib, which has demonstrated promising results in Phase II trials for platinum-sensitive recurrent ovarian cancer (Wu et al., 2022). Together, these studies firmly established PARPi as effective targeted therapies in ovarian cancer, especially for BRCA-mutated and HRD-positive patients.

Beyond ovarian cancer, PARPi have demonstrated efficacy in multiple BRCA-associated malignancies. In prostate cancer, the PROfound trial demonstrated that Olaparib significantly improved PFS and OS in men with metastatic resistant prostate cancer (mCRPC) harbouring alterations in BRCA1, BRCA2, or ATM who had progressed on prior androgen-receptor-targeted therapy (de Bono et al., 2020). This led to FDA approval of Olaparib for BRCA1/2 or ATM mutated mCRPC in 2020. Rucaparib has also received accelerated approval for BRCA-mutated mCRPC based on the Phase II TRITON2 trial (Abida et al., 2020), whose results were confirmed in the Phase III TRITON3 study (Fizazi et al., 2023).

In pancreatic cancer, the POLO trial showed that maintenance therapy with Olaparib significantly prolonged PFS compared with placebo in patients with a germline BRCA mutation and metastatic pancreatic cancer that had not progressed during first-line platinum-based chemotherapy (Golan et al., 2019). This led to the approval of Olaparib as maintenance treatment for germline BRCA (gBRCA) metastatic pancreatic cancer in 2019, representing the first targeted therapy approved for this patient population.

In the context of triple-negative breast cancer (TNBC), the approval of PARPi represents a significant advance. Approximately 15%–20% of TNBC patients harbour germline BRCA mutations, making them candidates for PARPi therapy (Sharma et al., 2014). The OlympiAD trial demonstrated improved PFS with Olaparib compared to standard chemotherapy in HER2-negative metastatic breast cancer patients with gBRCA, including those with TNBC (Robson et al., 2017). Similarly, the EMBRACA trial showed improved outcomes with Talazoparib in this patient population (Litton et al., 2018). Both Olaparib and Talazoparib have been approved in this indication.

Several next-generation PARPi are in advanced clinical development, aiming to overcome resistance mechanisms and improve therapeutic index. Saruparib (AZD5305) is a potent, highly selective PARP1 inhibitor that demonstrates approximately 400-fold greater selectivity for PARP1 compared to PARP2, with optimised pharmacokinetics and reduced myelosuppression compared to first-generation agents (Illuzzi et al., 2022). The PETRA trial has shown promising activity in patients with HRR-mutated advanced solid tumours, including those who progressed on prior PARPi (Herencia-Ropero et al., 2024). Veliparib (ABT-888) has demonstrated efficacy in the Phase III BROCADE3 trial, where its addition to Carboplatin and Paclitaxel significantly improved PFS in patients with BRCA-mutated, HER2-negative advanced breast cancer (Dieras et al., 2020). These next-generation agents may offer advantages in terms of central nervous system penetration, combination potential, and activity in patients with acquired resistance to current PARPi (Ngoi et al., 2021).

Beyond PARPi, several other DDR-targeting agents have shown promise across multiple cancer types, as described in Table 2. ATR and CHK1 inhibitors (ATRi and CHKi) represent a major class targeting replication stress vulnerabilities. ATR kinase serves as a critical sensor of replication stress, activating the CHK1-mediated S-phase checkpoint to prevent replication fork collapse (Cimprich and Cortez, 2008). Many TNBC cells demonstrate heightened replication stress due to oncogene activation, making them particularly dependent on the ATR-CHK1 pathway for prevention of fork collapse (Ma et al., 2012). Similar replication stress dependencies have been observed across diverse cancer types, including MYC-driven lymphomas, RAS-driven cancers, glioblastomas and malignant melanomas (Murga et al., 2011; Eich et al., 2013; Gilad et al., 2010). ATRi such as Berzosertib (VX-970) and CHK1ilike Prexasertib (LY2606368) have shown promising preclinical activity in tumours with high replication stress, both as monotherapy and in combination with DNA-damaging agents or radiotherapy (Hall et al., 2014; King et al., 2015). Furthermore, several ATRi have progressed to clinical evaluation, showing emerging activity as single agents in heavily pretreated ovarian cancers and in combination strategies designed to exploit replication stress or overcome resistance to PARPi. Notable examples include Berzosertib combined with Cisplatin in TNBC (Telli et al., 2022), Berzosertib plus Gemcitabine in platinum-resistant ovarian cancer (Konstantinopoulos et al., 2020), Berzosertib with Cisplatin and Gemcitabine in advanced urothelial carcinoma (Pal et al., 2021), and Ceralasertib combined with Durvalumab in immunotherapy-resistant melanoma (Kim et al., 2022), though dose-limiting myelosuppression remains a challenge (Ngoi et al., 2024).

**TABLE 2:** DDR-targeting agents beyond PARP inhibitors.

DDR Target	Agent	Cancer type/context	Treatment strategy	Evidence level	Key reference(s)
ATR	Berzosertib (VX-970)	Replication stress-high tumours	Monotherapy or + DNA-damaging agents/radiotherapy	Preclinical	Hall et al. (2014)
		TNBC	+ Cisplatin	Clinical trial (Phase I/II)	Telli et al. (2022)
		Platinum-resistant ovarian cancer	+ Gemcitabine	Clinical trial (Phase II)	Konstantinopoulos et al. (2020)
		Advanced urothelial carcinoma	+ Cisplatin + Gemcitabine	Clinical trial (Phase II)	Pal et al. (2021)
	Ceralasertib	Immunotherapy-resistant melanoma	+ Durvalumab	Clinical trial (Phase II)	Kim et al. (2022)
CHK1	Prexasertib (LY2606368)	Replication stress-high tumours	Monotherapy or + DNA-damaging agents/radiotherapy	Preclinical	King et al. (2015)
WEE1	Adavosertib (AZD1775)	Paediatric solid tumours	Monotherapy or + standard of care	Preclinical	Kolb et al. (2020)
		Pancreatic cancer	Monotherapy or + standard of care	Preclinical	Hartman et al. (2021)
		Lung cancer	Monotherapy or + standard of care	Preclinical	Richer et al. (2017)
		TP53-mutated ovarian cancer	Monotherapy or + chemotherapy (Carboplatin/Gemcitabine/Cisplatin)	Clinical trial (Phase I/II)	Oza et al. (2020)
		TP53- and RAS-mutant metastatic colorectal cancer	Monotherapy or + chemotherapy	Clinical trial (Phase I/II)	Seligmann et al. (2021)
		Head and neck squamous carcinoma	+ chemotherapy	Clinical trial (Phase I)	Mendez et al. (2018)
		Advanced solid tumours	Monotherapy or + chemotherapy	Clinical trial (Phase I/II)	Do et al. (2015)
DNA-PKcs	AZD7648	Various cancer types with defective HR	+Olaparib/radiotherapy/chemotherapy (Doxorubicin)	Preclinical	Fok et al. (2019)
	Peposertib (M3814)	Squamous cell head and neck and Non-small cell lung cancer xenografts	Radiotherapy	Preclinical	Zenke et al. (2020)
		Cervical cancer	Radiotherapy	Preclinical	Gordhandas et al. (2022)
		Advanced solid tumours	Monotherapy	Clinical trial (Phase I)	van Bussel et al. (2021)

Overview of DNA damage response (DDR) targeting agents in preclinical and clinical development, organized by molecular target. The table includes agents targeting ATR, CHK1, WEE1, and DNA-PKcs pathways. Evidence level distinguishes between preclinical studies and clinical trials with phase designation. Treatment strategies specify monotherapy or combination approaches with chemotherapy, radiotherapy, targeted therapy or immunotherapy. None of these agents have received regulatory approval to date.

WEE1 kinase represents another attractive DDR target, particularly in p53-deficient cancers. WEE1 normally prevents premature mitotic entry by phosphorylating and inactivating CDK1, allowing time for DNA repair completion (Parker and Piwnica-Worms, 1992). In p53-mutant cancers lacking G1 checkpoint control, cells become critically dependent on the G2/M checkpoint enforced by WEE1 (Leijen et al., 2010). The WEE1 inhibitor (WEE1i) Adavosertib (AZD1775) has demonstrated preclinical efficacy in multiple cancer types, including paediatric solid tumours (Kolb et al., 2020), pancreatic (Hartman et al., 2021), and lung cancers (Richer et al., 2017), both as monotherapy or in combination with the standard of care. Furthermore, several reports of ongoing clinical trials have confirmed the feasibility of targeting WEE1 in humans. Phase I and II trials of Adavosertib, either as monotherapy or combined with chemotherapy such as Carboplatin, Gemcitabine, or Cisplatin, have been conducted in TP53-mutated ovarian (Oza et al., 2020), TP53- and RAS-mutant metastatic colorectal (Seligmann et al., 2021), head and neck squamous carcinomas (Mendez et al., 2018) and other solid tumours (Do et al., 2015).

DNA-dependent protein kinase catalytic subunit (DNA-PKcs) is a central kinase in NHEJ, a DNA DSB repair pathway that becomes critical when HR is impaired (Hartley et al., 1995; Davis et al., 2014). Cancer cells with defective HR often rely on NHEJ to repair DSBs, creating a therapeutic vulnerability that can be exploited pharmacologically (Pearl et al., 2015). Several selective DNA-PKcs inhibitors have shown preclinical activity in combination with DNA-damaging agents, radiotherapy or chemotherapy (Gordhandas et al., 2022; Zenke et al., 2020; Fok et al., 2019). Early-phase clinical studies have begun to evaluate one of these agents, Peposertib (formerly M3814), as a single agent in advanced solid tumours, showing manageable toxicity and preliminary signs of activity (van Bussel et al., 2021).

In summary, while PARPi remain the only DDR-targeting agents that have received regulatory approval, their clinical success set the scene for thinking of DDR inhibition as a viable therapeutic strategy, validating the concept of synthetic lethality in oncology (Ngoi et al., 2022). Indeed, the inhibitors described above, such as those targeting ATR, CHK1, WEE1, and DNA-PKcs, are advancing through trials and highlight the broader potential of exploiting replication stress and checkpoint dependencies in cancer. Yet, these vulnerabilities do not exist in isolation and often emerge in the context of oncogene activation. In particular, the balance between MYC-driven genomic stress and compensatory repair mechanisms offers exploitable liabilities.

## 5 Combining MYC and DDR inhibitors in cancer therapy

The aggressive nature of cancer progression, characterised by rapid growth and proliferation, inevitably generates genomic instability and replication errors. In this context, MYC’s predominant role is damage mitigation, helping cancer cells repair the very stress that uncontrolled proliferation creates. The shift in balance towards MYC’s repair-promoting functions is a survival strategy for cancer cells but also their potential downfall (Doha and Sears, 2023).

In this final section, we examine the current preclinical literature, summarised in Table 3, where strategies to suppress MYC activity are combined with a range of DDR inhibitors, exploring therapeutic options that may be especially relevant in DDR-defective cancers or those with high replication stress.

**TABLE 3:** MYC inhibitors combined with ddr-targeting agents: preclinical evidence.

DR Target	DDR inhibitor	MYC inhibitor	MYC target type	Cancer type	Key reference(s)
PARP	Niraparib	Dinaciclib	Indirect (CDK)	TNBC	Carey et al. (2018)
	Talazoparib	Dinaciclib	Indirect (CDK)	TNBC	Baldwin et al. (2024)
	Olaparib	Omomyc	Direct	TNBC (PARPi-resistant)	Giuntini et al. (2025)
	Talazoparib	Omomyc	Direct	TNBC (PARPi-resistant)	Giuntini et al. (2025)
	Olaparib	JQ1	Indirect (BET)	Epithelial ovarian cancer (BRCA1/2 WT and PARPi-resistant)	Karakashev et al. (2017)
	PARP inhibitors	BRD4 inhibitors	Indirect (BET)	Multiple cancer types (HRD-independent)	Sun et al. (2018)
	Olaparib	PHA739358	Indirect (AURKA)	Neuroendocrine prostate cancer (MYCN-driven)	Zhang et al. (2018)
ATR	ATR inhibitors	AURKA inhibitors	Indirect (AURKA)	Neuroblastoma (MYCN-amplified)	Roeschert et al. (2021)
		BET inhibitors	Indirect (BET)	MYC-induced lymphoma	Muralidharan et al. (2016)
		BET inhibitors	Indirect (BET)	Melanoma	Muralidharan et al. (2017)
WEE1	AZD1775 (Adavosertib)	JQ1/AZD5153	Indirect (BET)	Non-small-cell lung cancer	Takashima et al. (2020)
		Panobinostat/Vorinostat	Indirect (HDAC)	Acute leukaemia (AZD1775-resistant)	Garcia et al. (2020)
		JQ1	Indirect (BET)	Acute leukaemia (AZD1775-resistant)	Garcia et al. (2020)

Preclinical combinations of MYC-targeting strategies with DDR inhibitors across multiple cancer types. MYC inhibition approaches are classified as direct (Omomyc) or indirect, with the latter including cyclin-dependent kinase (CDK), bromodomain and extra-terminal (BET), Aurora kinase A (AURKA), and histone deacetylase (HDAC) inhibitors. All evidence presented derives from preclinical studies including in vitro experiments, cell line-derived xenografts, and patient-derived xenograft models. Special disease contexts such as PARP inhibitor resistance, MYCN amplification, and drug-resistant settings are specified where relevant.

A leading example comes from TNBC. Compelling research suggests that MYC status may influence DDR pathway dependencies, creating synthetic lethal vulnerabilities that could be therapeutically exploited. Carey et al. demonstrated that combining MYC inhibition, albeit indirectly through Dinaciclib (a CDKi), with the PARPi Niraparib produced synergistic effects in TNBC models, independent of BRCA status. While neither Dinaciclib nor Niraparib are currently approved for TNBC treatment, these findings highlight the potential of targeting the MYC-DDR axis as a therapeutic strategy (Carey et al., 2018). More recently, Baldwin et al. reported that a nanoparticle formulation co-delivering Talazoparib and Dinaciclib further enhanced anti-tumour efficacy in TNBC cells and xenografts compared to either agent alone, supporting the therapeutic potential of this axis (Baldwin et al., 2024). Our recent study represents the first preclinical demonstration of combining a direct MYC inhibitor with PARP inhibition in TNBC (Giuntini et al., 2025). Using Omomyc, the first direct MYC inhibitor currently in Phase II clinical trials, this study reveals that MYC inhibition rapidly shuts down DDR gene expression in TNBC cells, inducing DNA damage and creating DDR deficiency. *In vitro* combinations of Omomyc with both Olaparib and Talazoparib (already approved in TNBC) demonstrated synergistic cytotoxicity. Remarkably, the therapeutic efficacy extended to PARPi-resistant settings, with *in vivo* studies across multiple PARPi-resistant cell line- and patient-derived xenografts showing that combined Omomyc and PARPi treatment resulted in significantly smaller tumours compared with either monotherapy. Furthermore, clinical sample analysis revealed that high MYC transcriptional activity serves as a predictive biomarker of PARPi resistance in TNBC patients, establishing a mechanistic rationale for patient stratification. This work provides compelling preclinical evidence that direct MYC inhibition can overcome intrinsic and acquired PARPi resistance, positioning the Omomyc-PARPi combination as a promising therapeutic strategy for TNBC patients who fail to respond to PARPi monotherapy (Giuntini et al., 2025). However, it remains premature to conclude that direct MYC inhibition will broadly overcome clinical PARPi resistance; this possibility requires controlled early-phase combination trials to evaluate safety, tolerability, biomarkers and mechanisms of resistance.

BET (bromodomain and extra-terminal) inhibitors (BETi) represent another promising, albeit indirect, approach for MYC suppression, with compelling rationale for combination with PARPi. BET proteins, particularly BRD4, regulate MYC expression by binding to acetylated histones at the MYC promoter and super-enhancer regions (Delmore et al., 2011). Building on this mechanistic basis, preclinical studies have shown that BETi can enhance the efficacy of PARPi across multiple tumour types. In epithelial ovarian cancer, Karakashev et al. demonstrated that the BETi JQ1 synergised with the PARPi Olaparib in BRCA1/2 wild-type models, and that BETi could also re-sensitise PARPi-resistant BRCA-mutant cells by suppressing WEE1 and TOPBP1 expression (Karakashev et al., 2017). These findings indicate that BETi can both broaden the therapeutic reach of PARPi and overcome acquired resistance. Extending this principle beyond ovarian cancer, Sun et al. showed that BRD4 inhibition synergised with PARPi across diverse cancer lineages, independent of intrinsic HRD status, through repression of CtIP, a key mediator of DNA end resection and HR repair (Sun et al., 2018). Together, these studies demonstrate that BETi induces a therapeutically exploitable DNA repair deficiency and establish a mechanistic link between indirect MYC targeting and enhanced PARPi sensitivity. However, despite reaching clinical trials over the past decade, BETi have not achieved regulatory approval due to dose-limiting toxicities such as thrombocytopenia and pulmonary arterial hypertension, combined with unclear trial data regarding their efficacy and mechanism of action in the context of MYC modulation (Whitfield and Soucek, 2025). Current strategies to advance BETi toward clinical application focus on combination approaches with other agents, which may allow for lower doses and improved therapeutic windows while exploiting synergistic mechanisms that extend beyond MYC suppression alone (Wang et al., 2023).

Aurora kinase inhibition represents another indirect strategy to modulate MYC function, with particular relevance in MYCN-driven and neuroendocrine tumours. Aurora kinase A (AURKA) binds and stabilises MYCN, preventing its proteasomal degradation and sustaining oncogenic transcriptional programmes (Otto et al., 2009). Consequently, AURKA inhibitors indirectly destabilise MYCN and have shown potent antitumour activity in preclinical models of childhood neuroblastoma (Brockmann et al., 2013). Beyond MYCN destabilisation, AURKA inhibition also perturbs DNA damage responses, creating therapeutic opportunities for combination with DDR inhibitors. For example, Zhang et al. identified a MYCN–PARP–DDR signalling axis in neuroendocrine prostate cancer, demonstrating that MYCN transcriptionally activates PARP1, PARP2, and other DDR effectors. Targeting this pathway with the AURKA inhibitor PHA739358 in combination with Olaparib markedly suppressed tumour growth in both cell line derived and patient-derived xenograft models, underscoring a mechanistic and therapeutic convergence between AURKA and PARPi in MYCN-driven malignancies (Zhang et al., 2018). Complementary evidence from MYCN-amplified neuroblastoma models further supports combining AURKA inhibitors and DDR inhibitors. Indeed, Roeschert et al. reported that co-targeting AURKA and ATR amplifies replication stress and DNA damage, resulting in profound tumour regression without overt toxicity (Roeschert et al., 2021). Together, these findings position AURKA inhibition as a mechanistically distinct yet convergent approach within the broader MYC–DDR therapeutic landscape, particularly relevant to aggressive brain and neuroendocrine tumours characterised by elevated MYC activity and replication stress.

Building upon the concept of targeting replication stress established with AURKA–ATR combinations, ATRirepresent a particularly rational partner for MYC inhibitors. MYC-overexpressing cells exhibit heightened dependence on ATR–CHK1 signalling to resolve chronic replication stress and maintain genome integrity. In a foundational study, Murga et al. demonstrated that MYC-driven lymphomas are acutely sensitive to ATR or CHK1 inhibition, whereas KRAS-driven tumours without replication stress were unaffected. These findings established that ATR dependency is a hallmark of oncogene-induced replication stress in MYC-driven cancers (Murga et al., 2011). Subsequent studies have shown that combining ATR inhibition with indirect MYC suppression can induce synthetic lethality. Nilsson Lab first demonstrated that dual inhibition of BET proteins and ATR in MYC-induced lymphoma cells triggers pronounced DNA damage, apoptosis, and senescence, both *in vitro* and *in vivo*. Mechanistically, BETi suppresses transcriptional programs necessary for replication and DNA repair, increasing dependency on ATR-mediated stress responses, while ATR inhibition in this context precipitates replication fork collapse and cell death (Muralidharan et al., 2016). In a follow-up study, the same group extended these findings to solid tumours, reporting that combined BETi and ATRi potently suppresses tumour growth and induces apoptosis in patient-derived xenograft models of melanoma. Importantly, these effects extended to MYC-driven malignancies beyond haematological models, suggesting a broader therapeutic principle (Muralidharan et al., 2017). Together, these studies provide compelling preclinical evidence that dual targeting of MYC signalling, and ATR-mediated checkpoint control can selectively collapse the replication-stress tolerance of MYC-overexpressing cancers.

WEE1 kinase inhibition represents another strategy for exploiting replication stress in rapidly proliferating tumours, and recent studies have begun to uncover how MYC modulation interacts with the WEE1 DNA repair pathway. Takashima et al. demonstrated that combining the WEE1i AZD1775 with BET bromodomain inhibitors such as JQ1 or AZD5153 synergistically suppressed the growth of non-small-cell lung cancer models *in vitro* and *in vivo*. Mechanistically, BETi reduced expression of the NHEJ factors XRCC4 and SHLD1, leading to accumulation of DNA DSBs, and concurrently downregulated MYT1, thereby driving premature mitotic entry and mitotic catastrophe in the presence of WEE1 blockade (Takashima et al., 2020). Building on this, García et al. provided evidence that MYC expression itself can mediate resistance to WEE1i. In AZD1775-resistant acute leukaemia cell lines, MYC levels were elevated and contributed directly to drug tolerance. Pharmacological suppression of MYC activity through HDAC or BETi, using Panobinostat, Vorinostat, or JQ1, restored sensitivity to AZD1775 and re-induced DNA damage responses (Garcia et al., 2020). These findings highlight a reciprocal relationship between WEE1 and MYC signalling: while MYC overexpression can confer resistance to WEE1i, targeting MYC transcriptional activity can instead potentiate WEE1-driven cytotoxicity.

Lastly, DNA-PKcs inhibitors (DNA-PKcsi) represent a promising, albeit underexplored, avenue for combination strategies with MYC-targeted therapies. High MYC expression induces replication stress and DNA damage, creating a dependency on DNA repair pathways including NHEJ, for which DNA-PKcs is required. A pooled kinase shRNA screen identified PRKDC (encoding DNA-PKcs) as a synthetic lethal partner in MYC-overexpressing human lung fibroblasts, highlighting the vulnerability of cells with active MYC signalling to DNA-PKcs inhibition (Zhou et al., 2014). The recently developed DNA-PKcsi AZD7648 has shown potent and selective inhibition of DNA-PKcs and enhances the efficacy of DNA-damaging agents such as doxorubicin and PARPi in preclinical models (Fok et al., 2019). Together, these findings provide a rationale for exploring combinations of MYC inhibitors with DNA-PKcsi as a strategy to simultaneously suppress oncogenic signalling and the compensatory DNA repair pathways that support cancer cell survival.

### 5.1 Limitations and translational challenges

Despite encouraging preclinical findings, several key limitations and translational challenges must be addressed before MYC–DDR combinations can be widely tested in patients.

- Model dependence.

Most synergy data derive from cell lines, isogenic systems, and xenografts that may not fully capture tumour heterogeneity, microenvironmental effects, or immune-mediated responses.

ii. Overlapping toxicity and dosing constraints.

DDR inhibitors often share haematologic toxicities (anemia, neutropenia, thrombocytopenia), which may be exacerbated in combination with each other or with indirect MYC-targeting therapies (such as BETi). Intermittent dosing, sequential scheduling, and pharmacodynamic biomarker-guided dose adjustments are pragmatic strategies to mitigate toxicity and should be systematically evaluated preclinically.

iii. Adaptive resistance and cross-resistance mechanisms.

Cancer cells may adapt to MYC-targeted therapies, particularly in HR-deficient backgrounds, by engaging compensatory DNA repair pathways, activating alternative transcriptional programs, or modulating checkpoint responses, potentially reducing the efficacy of MYC–DDR combinations. Specifically, tumours may evade MYC–DDR interventions via restoration of HR, activation of fork-protection pathways, upregulation of alternative transcriptional programmes, or checkpoint adaptation (e.g., PLK1-driven Claspin degradation). Longitudinal *in vitro* and *in vivo* evolution experiments, coupled with deep sequencing and functional HR assays, could be useful to define common escape routes and the impact of MYC inhibition in diverse genomic contexts.

iv. Translational trial design and biomarker development.

Early clinical development of MYC–DDR combination strategies require systematic preclinical safety validation, particularly regarding hematologic toxicity, which represents the dose-limiting adverse effect for many DDR-targeting agents. Rigorous *ex vivo* assays using human hematopoietic specimens could be well suited to quantify effects on progenitor viability, lineage-specific colony formation, and DNA-damage induction in normal cells, providing critical information for defining safe clinical starting doses and dose ratios. In parallel, trial design should address the practical measurement and clinical implementation of MYC as a predictive biomarker. Multiple assay platforms can quantify MYC activity: transcriptional signatures derived from RNA sequencing or NanoString panels measuring MYC target gene expression; immunohistochemistry for MYC protein nuclear accumulation; gene copy number assessment via FISH or next-generation sequencing panels. Critically, MYC biomarker assessment should be integrated with established HRD testing and BRCA mutation analysis, as these parameters may interact to determine optimal DDR inhibitor selection. For instance, MYC-high/HRD tumours may particularly benefit from these combinations, whereas MYC-high/HR-proficient tumours might require alternative/additional targeting strategies.

## 6 Conclusion

In this review, we have summarised the complex and paradoxical roles of MYC in the DNA damage response: as both an instigator of genomic instability and a promoter of compensatory DNA repair mechanisms. In normal physiology and early transformation, MYC’s activity accelerates replication and transcriptional programmes, imposing stress on the genome. Yet, in the cancer cell context, MYC overexpression drives a profound rewiring toward survival, directly inducing DNA repair pathways. Hence, reducing MYC activity limits the cell’s ability to cope with replication stress, and when this is coupled with inhibition of key DNA repair pathways such as ATR/CHK1, WEE1, DNA-PKcs, or PARP, cancer cells can be pushed toward irreversible genomic instability.

We have described in detail the preclinical studies, including combinations of MYC suppression with PARPi, ATRi, CHK1i, or WEE1i, which provide proof-of-concept for this approach. These findings highlight that MYC-driven tumours possess distinct dependencies on specific DNA repair mechanisms that can be therapeutically exploited.

Equally transformative is the changing perception of MYC inhibition itself. Once deemed impossible, direct MYC inhibition is now advancing in the clinic (Whitfield and Soucek, 2025). The dominant-negative MYC inhibitor Omomyc (a.k.a OMO-103 in its first clinical incarnation) has completed a first-in-human Phase I trial, demonstrating excellent safety and clinical activity (Garralda et al., 2024), and is now progressing through Phase Ib and II trials.

Following this clinical milestone, in Giuntini et al. we report the first study where a direct MYC inhibitor is combined with a DDR-targeting agent (Giuntini et al., 2025). This work provides experimental evidence that pharmacological MYC blockade can be effectively integrated with PARPi, enhancing antitumour activity in preclinical models. Importantly, the investigation was conducted using two clinically approved PARPi (Olaparib and Talazoparib) in TNBC. Overall, this study establishes a clear functional connection between MYC suppression and DDR pathway targeting, offering a concrete example of how direct MYC inhibitors can potentiate existing therapeutic strategies that exploit replication stress and repair dependency in cancer cells.

Looking forward, compelling opportunities lie in expanding dual targeting of MYC and the DDR. Preclinical studies should focus on testing direct MYC inhibition in combination with a wider range of DDR-targeting agents, including ATR/CHK1, WEE1, and DNA-PKcs inhibitors. These studies will help define highly effective combinations, optimise dosing and identify biomarkers of MYC dependency and DNA repair vulnerability. By exploring diverse tumour models, preclinical work can prioritise strategies with the strongest translational potential.

Clinically, early trials could leverage the momentum of OMO-103 and approved PARPi, initially focusing on patients with BRCA-mutated or HRD tumours, where mechanistic rationale and preliminary data are strongest. Incorporating comprehensive biomarker assessments of MYC activity (as detailed in Section 5.1), integrated with homologous recombination deficiency and BRCA mutation status, will be essential to guide patient selection, monitor response, and refine combination strategies. As safety and efficacy data are gathered, these trials can expand to additional DDR inhibitors and tumour types, translating mechanistic insights into broader clinical benefit.

Together, this approach writes a clear script for future studies: preclinical exploration defines the limits and mechanisms of vulnerability, while clinical studies test their applicability and impact in patients. The combination of direct MYC inhibition with DDR-targeting agents now moves from conceptual rationale to a concrete translational opportunity, preparing the stage for this novel therapeutic duo across multiple cancer contexts.
